# Spatial-temporal graph neural networks for groundwater data

**DOI:** 10.1038/s41598-024-75385-2

**Published:** 2024-10-19

**Authors:** Maria Luisa Taccari, He Wang, Jonathan Nuttall, Xiaohui Chen, Peter K. Jimack

**Affiliations:** 1 School of Civil Engineering, University of Leeds, Leeds, UK; 2 Forecast Department, European Centre for Medium-Range Weather Forecasts (ECMWF), Reading, UK; 3 Centre for Artificial Intelligence, Department of Computer Science, University College London, London, UK; 4 Deltares, Delft, The Netherlands; 5 School of Computing, University of Leeds, Leeds, UK

**Keywords:** Graph neural networks, Groundwater levels, Surrogate modeling, Deep learning, Civil engineering, Computational science

## Abstract

This paper introduces a novel application of spatial-temporal graph neural networks (ST-GNNs) to predict groundwater levels. Groundwater level prediction is inherently complex, influenced by various hydrological, meteorological, and anthropogenic factors. Traditional prediction models often struggle with the nonlinearity and non-stationary characteristics of groundwater data. Our study leverages the capabilities of ST-GNNs to address these challenges in the Overbetuwe area, Netherlands. We utilize a comprehensive dataset encompassing 395 groundwater level time series and auxiliary data such as precipitation, evaporation, river stages, and pumping well data. The graph-based framework of our ST-GNN model facilitates the integration of spatial interconnectivity and temporal dynamics, capturing the complex interactions within the groundwater system. Our modified Multivariate Time Graph Neural Network model shows significant improvements over traditional methods, particularly in handling missing data and forecasting future groundwater levels with minimal bias. The model’s performance is rigorously evaluated when trained and applied with both synthetic and measured data, demonstrating superior accuracy and robustness in comparison to traditional numerical models in long-term forecasting. The study’s findings highlight the potential of ST-GNNs in environmental modeling, offering a significant step forward in predictive modeling of groundwater levels.

## Introduction

The complexity of groundwater level (GWL) prediction and modeling arises from its nonlinearity and sensitivity to various hydrological, meteorological, and anthropogenic influences^1–3^. Conventional GWL models, such as physical-based and traditional statistical methods, are limited by their high parameterization needs. This requirement makes the models computationally difficult to calibrate, as each parameter must be accurately estimated to reflect real-world conditions. Additionally, the models are challenged by computational intensity and difficulty in capturing the temporal evolution and nonlinearity in GWL data^4–7^. In this context, deep learning models and, in particular, spatial-temporal graph neural networks (ST-GNNs) offer promising new avenues for accurate and efficient groundwater forecasting. Our study focuses on leveraging ST-GNNs to predict groundwater levels in the Overbetuwe area, Netherlands. The selection of this area is driven by the availability of data^8^.

Machine learning methods, particularly data-driven ones, have been successfully applied in groundwater level prediction studies without requiring detailed physical process knowledge^9–11^. Recent works have observed an evolving landscape in groundwater level forecasting, shifting from conventional shallow networks to deep learning techniques^12^. Unlike physically-based models, which usually require a detailed understanding of local conditions and extensive calibration, data-driven models such as artificial neural networks can predict target variables using only relevant driving forces. LSTMs, designed for long-term time series data prediction, are equipped with memory cells capable of retaining critical information about historical events, making them well-suited for extracting non-linear spatio-temporal groundwater patterns. Zhang et al.’s study employing LSTM models to simulate water table fluctuations using a range of inputs over 14 years showed extremely high accuracy, illustrating the potential of these models to outperform traditional ANN approaches^13^. A previous study has instead shown that 1D-convolutional neural networks (CNNs) are a good choice for groundwater level simulation, as they outperform LSTM models in terms of accuracy and calculation speed.^10^ However, these methods focus predominantly on temporal dynamics and often overlook the spatial relationships in GWL data, a gap that ST-GNNs are well-equipped to address. This gap in research is where this paper contributes.

ST-GNNs, rooted in graph theory and neural network methodologies, are particularly effective in datasets where spatial interconnectivity and temporal changes are significant. The ST-GNNs process inputs consisting of multivariate time series data, accompanied by a graph structure which delineates the interconnections between the variables within the multivariate time series. In these networks, spatial correlations among nodes are effectively represented through graph convolution techniques, while the temporal relations among past states are analyzed using recurrent neural network architectures^14–21^

While ST-GNNs have primarily been used in traffic prediction and skeleton-based action recognition, their application has recently extended to broader scientific fields, including meteorology^22,23^ and seismology^24^. This study harnesses the power of ST-GNNs to predict GWL in the Overbetuwe area, Netherlands. Unlike traditional methods, ST-GNNs adeptly handle unstructured spatial data and capture the complex interplay of spatial and temporal dynamics affecting GWL. The selection of ST-GNNs for this task is motivated by the distinct characteristics of the data and the specific challenges they introduce to modeling. Firstly, the nature of groundwater data, characterized by its complex spatial and temporal interactions, aligns well with the capabilities of spatial-temporal graph modeling. This approach offers the necessary granularity and flexibility to accurately model the intricate relationships within multivariate time series data. Secondly, the data encountered in this study is inherently hybrid, containing both continuous and discrete elements. This heterogeneity requires a modeling solution that can adeptly manage such diverse data types. Furthermore, the data’s noisiness and sparsity, spanning both spatial and temporal dimensions, pose significant challenges^25^. These conditions necessitate a modeling strategy capable of discerning correlations between observations at various locations and times, yet the reliance on black-box learning methods is constrained by the limited quantity of data available. Therefore, the deployment of ST-GNN, infused with a degree of prior knowledge, emerges as a requisite strategy. By incorporating control variables like pumping rates and considering multiple aquifers, the proposed ST-GNN model extends the applicability of GNNs in hydrological forecasting beyond current capabilities, addressing challenges such as missing data and ensuring model generalizability.

One recent study that does address spatial relationships in groundwater forecasting is by Bai et al.^26^. In their work, they employ a graph neural network to forecast groundwater dynamics in the southwest area of British Columbia, Canada. Bai et al.’s model demonstrates superior performance compared to baseline models like LSTM^27^ and Gated recurrent unit (GRU)^28^. However, there are notable differences between Bai et al.’s approach and our study. While Bai et al. focus on groundwater dynamics in a region predominantly influenced by rainfall and characterized by high seasonality in groundwater level (GWL) fluctuations, our research extends to the Overbetuwe area, Netherlands, which does not exhibit such pronounced seasonal patterns. Our study area is influenced by a broader range of hydrological, meteorological, and anthropogenic factors, including the effects of pumping wells and river stages, contributing to more complex and nuanced groundwater behavior. Moreover, our choices of GNN architectures and predefined adjacency matrix contrast with Bai et al.’s use of Graph WaveNet and a self-adaptive adjacency matrix. Our model is tailored to our unique dataset and the specific challenges it presents, including the integration of control variables such as pumping rates, and the definition of the graph structure to represent the differet hydrological connections. Lastly, a critical advancement in our methodology, compared to Bai et al., is our approach to handling data gaps. Bai et al. exclude wells with gaps larger than one month and interpolate smaller gaps, potentially overlooking valuable information. Conversely, we introduce a masking strategy to track the locations and times of missing values, seamlessly integrating this information into our loss function to avoid training on these gaps, thereby enhancing model accuracy and reliability.

This study goes beyond traditional forecasting by exploring the ST-GNN model’s utility in scenario planning and decision-making for drinking water extraction, considering varying weather condition scenarios months in advance. This capability is pivotal for managing water resources sustainably, ensuring that the extraction volumes are optimized to prevent drought conditions. Furthermore, the deep learning model supports rapid computational capabilities. This feature enables the quick generation and simulation of multiple scenarios, making it an invaluable resource for strategic planning and promoting efficient water usage.

The paper is structured as follows: following the introduction, we describe our methodology, including data description, preprocessing, and model architecture in Section 2. Section 3 presents the results, including an abation study and a comparative analysis with the state-of-the-art traditional numerical model. Finally, we conclude in Section 4 with a discussion on the implications of our findings for hydrological forecasting and future research directions.

## Methodology

This section outlines the methodology for predicting groundwater levels using ST-GNNs. It starts with a detailed description of data from the Overbetuwe area in the Netherlands, focusing on hydrological, meteorological, and anthropogenic factors affecting groundwater levels. The preprocessing steps for model training are then presented, followed by an explanation of a graph-based framework designed to capture the complex spatial-temporal relationships in groundwater data. Finally, it discusses training strategies and error metrics to optimize model performance and accurately evaluate its predictive capabilities.

### Data description

The study focuses on the Overbetuwe area in the Netherlands, a polder region approximately 30 km by 10 km in size, flanked by two branches of the Rhine river. The land surface elevation varies from around +10 m NAP (Amsterdam Ordnance Datum) in the east to around +7 m NAP in the west. The shallow subsurface is characterized by a low-permeable phreatic layer, underlain by two aquifers separated by an aquitard. The groundwater is relatively shallow, with the depth to the water table varying between 0.8 and 4.2 m. The near-surface layer features a low-permeability phreatic zone composed mainly of clay and sandy clay. Beneath this zone lie two aquifers, which are divided by an aquitard. This aquitard is made up of clay, with its thickness ranging from 0 to 15 meters^8^. For a more comprehensive understanding of the hydrological system, the reader is referred to the work of Brakenhoff et al^8^.

**Fig. 1:**
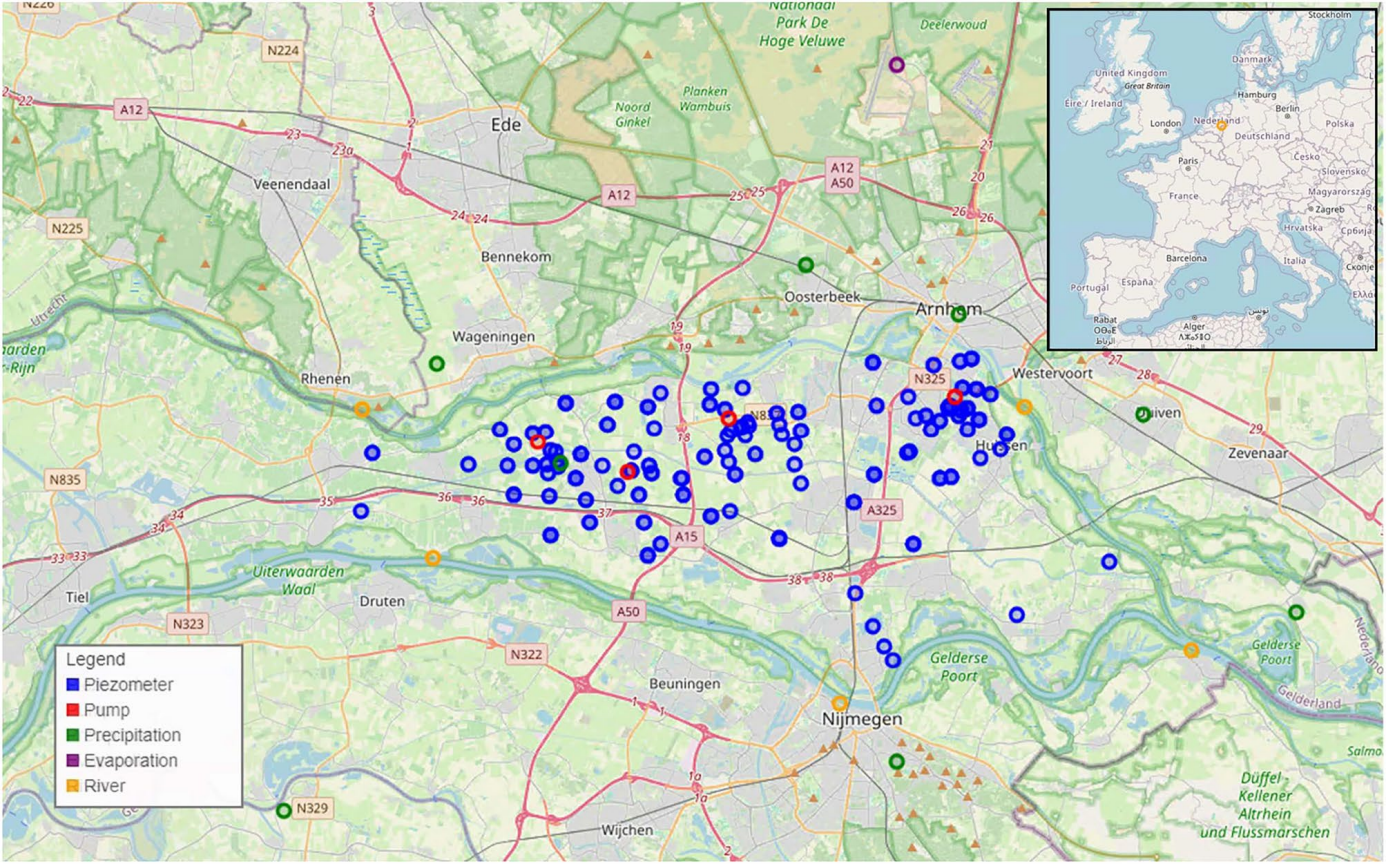
Illustration of the sensor network. Different types of sensors are represented by distinct colors, providing an overview of the spatial distribution and categorization of each sensor within the study area. Notably, one evaporation sensor is located outside the depicted map area. Additionally, the precise locations of some observation points may not be distinctly visible, as certain sensors share the same coordinates but are situated at different depths, and others are too closely positioned to be distinguished on the map. The underlying map data is $\copyright$ OpenStreetMap contributors and is available under the Open Database License (ODbL)^29^.

The study encompasses data from 213 observation wells, also called piezometers, yielding a total of 395 groundwater level time series. Some of these time series originate from the same geographical location (but differing in terms of depth). While each time series begins in a different year, some starting as early as the 1950s, the frequency and availability of data have evolved significantly over time. Initially, the data collection for these time series was on a monthly basis. However, the introduction of automatic loggers at the turn of the new millennium enabled an increase in frequency to daily or even more frequent recordings. Due to the higher frequency and availability of modern data, we preferred to use these for a weekly analysis to ensure more consistent and detailed temporal coverage. Figure 1 visually showcases the distribution and types of sensors across the Overbetuwe area.

In addition to the groundwater levels from the observation wells, which represent the primary variable of interest, other types of time series data are recorded as exogenous variables: precipitation, evaporation, river stages, and pumping wells. Of these, only the pumping well serves as the control variable. Daily precipitation data is available from seven measurement stations, and evaporation data from two weather stations. Additionally, river stage measurements are taken every 10 minutes, and drinking water extraction data is gathered daily from 4 stations. The metadata for each of these time series includes location coordinates and depth where applicable.

### Data preprocessing

In addressing the critical challenges of data sparsity and noise inherent in the dataset, the preprocessing strategy lays a foundational stone for the subsequent analysis and modeling phases of this study. The complexity introduced by noisy data and sparse datasets-characterized by significant gaps and intermittent recordings-necessitate a rigorous approach to data cleaning and selection. Refining the dataset to mitigate these challenges, enhances the reliability of the analysis and establishes a robust basis for the modeling phase. The approach to preprocessing not only addresses the immediate issues of data quality and completeness but also serves as a crucial contribution of this study, illustrating the significance of a well-considered preprocessing phase for robust forecasting results.

The starting point for this analysis is established as the year 2004. This choice rests on two key factors: the comprehensive availability of time series data from observation wells starting from this year, and the significant portion of these data recorded on at least a daily basis. To balance maintaining sufficient data and managing its variability, and considering the relatively slow daily variations, the data frequency is adjusted to a weekly basis, with values averaged.

Out of the 395 available time series, 200 are selected, prioritizing those with the most complete data. The excluded time series had an exceedingly high amount of missing data points, corresponding to more than 20% of their data missing. Initially, the overall percentage of missing values for the observation wells stands at 29.3%; this selection reduces the overall percentage of missing values to 8.1%. Missing values undergo linear interpolation, and a mask is introduced to track the locations and times of these missing values. This mask proves instrumental in the loss function, ensuring that these points are not used for training. The purpose of the linear interpolation is to provide a continuous dataset for model input, while the masking ensures that only real data points are used in the training loss function, maintaining the integrity and accuracy of the training process. For the river stage data, only five locations with no missing data are retained: Dodewaard, IJsselkop, Nijmegen haven, Grebbe, and Pannerdense Kop. The other types of measurements exhibit no missing data issues.

All data are rescaled within the range (0, 1).The dataset is chronologically divided, with 80% allocated for training starting from 2004-01-04, and 10% reserved for validation. However, using a separate 10% for testing resulted in overconfident and unrepresentative outcomes. To address this, the validation and test sets were combined, leading to the use of the full 20% of the data, starting from 2018-04-01, for testing purposes. This adjustment provided a larger and more representative test set, which is particularly relevant for assessing the model’s performance on more recent data. While this combined set does not include a separate validation set, it effectively maximizes the use of available data given the constraints of our limited dataset.

### Graph-based framework in groundwater level forecasting

In this study, a deep learning graph-based approach is adopted to forecast groundwater levels in the Overbetuwe area. Each measurement, whether from observation wells (the subjects of the forecasting task), precipitation, or other hydrological data points, represents a node in a graph. The values at each time step $t$ are denoted by $\textbf{z}_t \in \textbf{R}^{N'}$, where $N'$ is the number of time series of groundwater levels and $z_{t}[i] \in \mathbb {R}$ represents the specific measurement value at that time step.

**Objective** The primary goal is to predict future groundwater levels using historical observations within an input window of $W$ time steps, leading up to time step $P$. This historical data is represented as $\textbf{X} = \{\textbf{z}_{t_{P-W+1}}, \textbf{z}_{t_{P-W+2}}, \ldots , \textbf{z}_{t_P}\}$. The aim is to forecast a sequence of future values over a forecasting window of $Q$ time steps, denoted as $\textbf{Y} = \{\textbf{z}_{t_{P+1}}, \textbf{z}_{t_{P+2}}, \ldots , \textbf{z}_{t_{P+Q}}\}$.

**Incorporating Auxiliary Data** To enhance the model’s predictive capability, auxiliary features are integrated including precipitation, evaporation, river stages, and pumping well data, with their number of time series being $N''$. These features are considered up to and including the forecasting window $t_{P+Q}$: $\mathbf {X'} = \{\mathbf {s'}_{t_{P-W+1}}, \mathbf {s'}_{t_{P-W+2}}, \ldots , \mathbf {s'}_{t_{P+Q}}\}$, where each $\mathbf {s'}_{t_i} \in \textbf{R}^{N''}$ contains the auxiliary data at time step $t_i$. This auxiliary dataset $\mathbf {X'}$ then concatenates with the historical input data $\textbf{X}$, which only includes observations from time step $t_{P-W+1}$ to $t_P$. Padding applies to align the different lengths of historical and auxiliary data for concatenation. In practical applications, while exogenous variables such as precipitation and river stages will be forecasted (or modelled), the pumping well data is treated as a controllable variable. The approach constructs a mapping function $f: (\textbf{X}, \mathbf {X'}) \rightarrow \textbf{Y}$, where $\textbf{X}$ includes the historical data up to time step $t_P$, and $\mathbf {X'}$ comprises the extended auxiliary data up to $t_{P+Q}$, with the goal of predicting future values $\textbf{Y}$. The assumption of deterministic forecasts for exogenous variables is made for several reasons. Firstly, forecast data for those periods are not readily available to us. Secondly, using deterministic inputs simplifies the initial modeling process by allowing the concatenation of historical and future data without introducing the complexities of forecast uncertainty. Additionally, this design choice provides a robust framework for conducting ensemble, sensitivity, and scenario analyses.

**Graph Definition**

This study conceptualizes a graph $G = (V, E)$ to represent the hydrological system. $V$ signifies the set of nodes, each corresponding to a distinct hydrological measurement, while $E$ encapsulates the connections or relationships among these measurements. The total number of nodes in the graph $N$ is the sum of two subsets: $N'$ representing groundwater level measurements and $N''$ comprising other hydrological factors. Forecasting efforts focus on $N'$, utilizing both historical groundwater levels and various exogenous variables.

For each node $v \in V$, corresponding to a specific hydrological measurement or time series, its neighborhood $N(v)$ is defined. This neighborhood consists of other measurements that are hydrologically interconnected or influenced by $v$. Each piezometer is linked to its three closest counterparts, determined by Euclidean distance, a choice that balances the graph’s connectivity without making it too sparse or overly dense, as established through an ablation study presented in 3.1. Furthermore, each piezometer establishes connections with all pumping locations, the nearest precipitation and evaporation stations, and the two closest river measurement points. The adjacency matrix $\textbf{A} \in \mathbb {R}^{N \times N}$ where $N$=$N'$+$N''$ represents these hydrological connections. In this matrix, an entry $A_{ij}$ assigns a non-zero value if there is a connection between nodes $i$ and $j$, and zero if no such relationship exists. The assigned values in the adjacency matrix, ranging from 0.1 to 0.5, differentiate the types of hydrological connections, such as those between piezometers, between piezometers and pumping wells, and so on. Currently, no explicit geological variables are included in the model; instead, the graph structure is designed to capture spatial relationships indirectly through the connections between nodes. Future work could investigate incorporating explicit geological variables, such as aquifer properties, as edge weights to potentially enhance the model’s performance. As the study area increases, the number of nodes in the graph $N$ will also increase, resulting in higher computational costs due to the increased complexity and size of the adjacency matrix A. In larger study areas, it might be more efficient to construct sparser graphs by reducing the number of connections per node. Sparse graphs could still capture the essential hydrological relationships without overwhelming the computational capacity, thus providing a balanced trade-off between accuracy and efficiency.

### Model architecture

A modified version of the Multivariate Time Graph Neural Network (MTGNN) model, originally described by^14^, is adapted in this study. This adaptation omits the graph learning layer, favoring predefined graph structures which we found to yield significantly improved results.

**Fig. 2:**
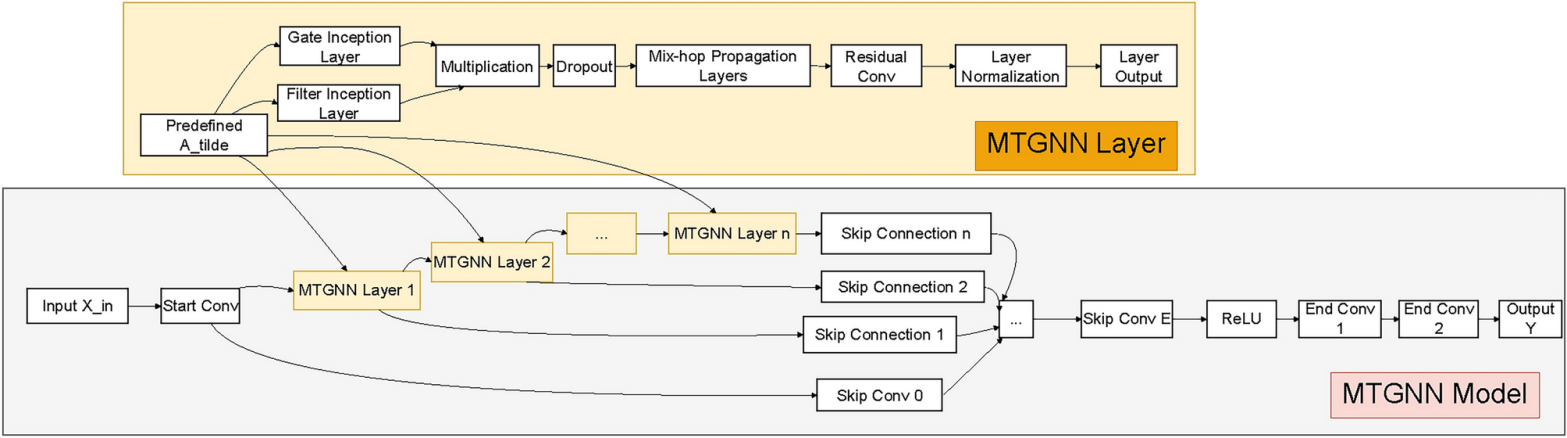
The model architecture includes both graph and temporal convolution modules, highlighted by their sequential processing and integration of skip connections to facilitate data flow and information preservation across layers.

The architecture, visualized in Fig. 2, demonstrates the sequential processing of data through the model. Input data is first subjected to a starting convolution operation, after which it progresses through multiple graph and temporal convolution modules, designed to capture spatial and temporal dependencies, respectively. These modules are interconnected by skip connections, enhancing the model’s ability to preserve information across layers. The graph convolution module operates on spatial relationships by aggregating node information with neighboring nodes, leveraging mix-hop propagation layers for this purpose. Meanwhile, the temporal convolution module employs dilated 1D convolution filters to analyze temporal patterns, utilizing a combination of filter and gating layers to modulate the flow of information. Finally, the output module itself consists of two 1x1 convolution layers, adjusting the channel dimension of the input to meet the desired output dimension.

For a more detailed description of the architecture, readers are referred to the original article^14^, while the subsequent sections of this paper will only briefly present the temporal and spatial modules, highlighting the innovations in this work.

#### Graph convolution module

The graph convolution modules address spatial dependencies by aggregating information from each node with its neighbors. This involves two mix-hop propagation layers, which facilitate the inflow and outflow of information at each node^14^. The mix-hop propagation layer, given by Equation 1 and Equation 2, handles the spatial information flow across nodes in the network:

1 $$\begin{aligned} \textbf{H}^{(k)}= & \beta \textbf{H}_{in} + (1 - \beta ) \tilde{\textbf{A}} \textbf{H}^{(k-1)}, \end{aligned}$$

2 $$\begin{aligned} \textbf{H}_{out}= & \sum _{k=0}^{K} \textbf{H}^{(k)} \textbf{W}^{(k)}. \end{aligned}$$

In Equation 1, $\textbf{H}^{(k)}$ represents the hidden states at the *k*-th propagation step, $\beta$ is a hyperparameter controlling the retention ratio of the root node’s original states, $\textbf{H}_{in}$ denotes the input hidden states from the previous layer, $\tilde{\textbf{A}}$ is the normalized adjacency matrix including self-connections, and $\textbf{H}^{(k-1)}$ are the hidden states from the $(k-1)$-th propagation step. In Equation 2, $\textbf{H}_{out}$ represents the output hidden states of the current layer, *K* is the depth of propagation, and $\textbf{W}^{(k)}$ is the parameter matrix acting as a feature selector at each propagation step.

#### Temporal convolution module

The temporal convolution modules focus on capturing the temporal dynamics within the data. These modules utilize standard dilated 1D convolution filters, arranged in two layers: a filter layer followed by a tanh activation function and a gating layer with a sigmoid activation function^14^. The temporal convolution module utilizes dilated 1D convolution filters:

3 $$\begin{aligned} \textbf{z} = \text {concat}(\textbf{z} \star \textbf{f}_{1 \times 1}, \textbf{z} \star \textbf{f}_{1 \times 2}), \end{aligned}$$

where $\textbf{z}$ is the input 1D sequence, $\textbf{f}_{1 \times k}$ are the convolution filters of varying sizes and $\textbf{z} \star \textbf{f}_{1 \times k}$ represents the dilated convolution operation. This study deviates from the original model’s filter sizes of $1 \times 2$, $1 \times 3$, $1 \times 6$, and $1 \times 7$, whose combinations were designed to capture a variety of inherent periods typical of temporal signals. Given our data is measured in weeks without significant seasonal fluctuations, this broad spectrum of filter sizes was not deemed necessary and the choice of the filters is based on an ablation study presented in 3.1. Dilated convolution introduces “gaps” in the convolution kernel to extend its coverage on the input feature map without increasing parameters or computation. This efficiently broadens the receptive field, enabling the incorporation of wider input information without escalating the model’s parameter count. The outputs of convolution operations across different filter sizes are concatenated by the $\text {concat}$ function. Finally, the skip connection layer and output module, which standardize and transform the data for final output, follow the temporal and spatial modules.

### Training approach

Our model harnesses historical observations to forecast future groundwater levels. It adopts a recursive forecasting approach, wherein the groundwater level prediction at time $t+1$ serves as an input for the subsequent time step $t+2$, thereby recursively using forecasted outputs as historical inputs when the prediction window exceeds a single time step. This approach is applied consistently during both training and testing phases. This recursive strategy is enhanced by incorporating exogenous variables, which, although they might originate from forecasts of different models or be based on scenarios to be considered, are given as inputs for the next time step without being forecasted themselves. Groundwater management entities need to determine the amount of water to extract and assess the corresponding impacts, making the pumping rate a controllable variable rather than one subject to forecasting. This distinction is crucial because the pumping rate is based on strategic decisions, whereas other exogenous variables such as precipitation and evaporation are subject to forecast uncertainties. By assuming deterministic forecasts for these variables, it enables the user to conduct ensemble, sensitivity, and scenario analyses, providing a comprehensive assessment of the model’s robustness and performance under various conditions.

Furthermore, the training regime involves adjusting the size of the prediction window dynamically, thus tailoring the model for different forecasting scenarios.

The training employs an iterative process that gradually extends the prediction window. Specifically, our model utilizes a past window size $W$ of 5 time steps, with each time step representing a week, to incorporate recent observations and a future prediction window $F$ of up to 8 time steps. For the ablation study, training is confined to forecasts up to only 2 weeks ahead to expedite the processing of numerous model configurations. During testing, the model extends its forecasting to a future prediction window F of up to 100 weeks to assess long-term predictive capabilities. Through this methodology, the model concurrently optimizes forecasts across the entire prediction horizon during training, ensuring a comprehensive learning process. During inference, exogenous variables are provided as actual values rather than forecasts, avoiding forecast errors and relying solely on previous groundwater level predictions. This justifies extending the prediction window to 100 weeks, as it remains practical for long-term planning and allows for the assessment of error propagation.

The loss function, as defined in Equation 4, is designed to accommodate missing data within the time series through a masked mean squared error method. The $\text {mask}_{i, t}$ is a binary indicator where the value is set to 0 when data is missing and 1 otherwise. This binary mask ensures that while the missing points are linearly interpolated and used as inputs, they do not contribute to the loss calculation, thereby preserving the integrity of the model’s training process. In this way, the model concentrates on accurately predicting the available data points, while effectively disregarding the segments with missing or unreliable data.

4 $$\begin{aligned} \text {Loss} = \sum _{i=1}^{B} \sum _{t=1}^{F} \text {mask}_{i, t} \cdot (y_{i, t} - \hat{y}_{i, t})^2, \end{aligned}$$

Here, $B$ represents the batch size and $F$ is the length of the future prediction window. The actual observed values are denoted by $y_{i, t}$, and the model’s predicted values are represented by $\hat{y}_{i, t}$. The use of the mask in the loss function ensures that the model is trained primarily on the robust data points, providing a reliable performance metric that truly reflects the model’s forecasting capabilities.

To quantify the accuracy of our forecasting model, we utilize the Root Mean Square Error (RMSE) as the primary error metrics. The RMSE, detailed in Equation 5, quantifies the forecast error’s magnitude and indicates the average deviation between the model’s predictions and the actual observed values.

5 $$\begin{aligned} \text {RMSE} = \sqrt{\frac{1}{M}\sum _{i=1}^{M}(y_{i} - \hat{y}_{i})^2}, \end{aligned}$$

In Equation 5, $M$ denotes the number of forecasts, $y_{i}$ the actual observed values, and $\hat{y}_{i}$ the values predicted by the model. A lower RMSE value indicates a more accurate model.

## Results

This section presents a rigorous evaluation of the deep learning model’s efficacy in long-term groundwater level forecasting. The first part presents an ablation study, analyzing the influence of various model components and parameters on the forecasting results. Subsequently, the model’s efficacy is evaluated on real-world data, with performance comparisons drawn against the traditional numerical MODFLOW model.

### Ablation study

This section explores various model configurations and parameters with the goal of assessing the impact of these variables on forecasting accuracy, quantified by the Root Mean Square Error (RMSE). A key focus is the evaluation of spatial and temporal convolution modules, alongside the influence of graph parameters on model performance. In the ablation study, using a historical window size $W$ of 5 time steps, each corresponding to one week, training is confined to forecasts up to only 2 weeks ahead to expedite the processing of numerous model configurations. During testing, the model extends its forecasting to a future prediction window $F$ of 100 weeks.

To facilitate the ablation study and comprehensively assess model performance under controlled conditions, a synthetic dataset is employed. The synthetic dataset is derived from the MODFLOW groundwater model for the Overbetuwe area, a component of the larger MORIA model^30^, which represents a collaborative effort involving several organizations, including provinces, waterboards, and drinking water companies. The model leverages a comprehensive range of input data to simulate groundwater dynamics accurately, including subsurface schematization through REGIS II.2^30^. It accounts for the actual meteorological conditions and known groundwater abstractions during the period in question, facilitating a balanced comparison with the real-world data. Additionally, it incorporates factors like anisotropy, drainage, boundary conditions, initial heads, and storage coefficients. The dataset includes over 800 observation wells and spans from 2008 to 2019, with daily recordings. To ensure a consistent evaluation, the dataset consists of 188 observation wells that have both simulated and real-world data. The unavailability of simulated data for 12 out of the original 200 wells leads to selecting alternative wells with real data, which are not chosen among the real-world observations due to their higher rates of missing data. Although the dataset includes spatial coordinates, depth, and node type as static features, certain aspects like the top and bottom elevations of aquifers and hydraulic conductivity for aquifers are not directly used in model training. Instead, they are considered intrinsic properties learned by the GNN. Using synthetic data from a MODFLOW model in the ablation study helps understand the model’s performance and optimize its configuration before applying it to real-world data. However, this approach is not limited to areas with synthetic data or a MODFLOW model. In regions without such models, the methodology can proceed directly with observed data, maintaining the model’s robustness. Synthetic data may not inherently capture real-world changes in time or shifts in distribution unless explicitly modeled. For this reason, the model is retrained using real-world data to ensure its effectiveness under diverse conditions.

The analysis demonstrates a notable improvement in performance when using synthetic data (see below for details), for which the ST-GNN model secures a baseline RMSE of 8.13 cm, in contrast to RMSE of 16.9 cm obtained with real data. This underscores the utility of synthetic data in honing forecast accuracy and in refining the model parameters during the ablation study. The increased error observed with real data can likely be attributed to the inherent noise present in such real-world data. Subsequent studies could explore the estimation of this noise and its introduction into synthetic datasets to achieve a more authentic representation of measured data, particularly when such data are insufficient for training deep learning models.

Furthermore, the ablation study assesses the performance of different hyperparameters and model configurations, beginning with the examination of the utility of both the temporal and graph convolutional modules. Initially, replacing each dilated 1D convolution layer in the temporal module with an LSTM, followed by a fully connected layer, results in an increased error, raising the RMSE to 34.6 cm. Removing the graph convolution module from the model incurs a pronounced increase in RMSE to 31.0 cm as well, clearly illustrating the vital contribution of both modules to maintaining accuracy. This analysis focuses on modifying the internal structure of the ST-GNN to highlight the importance of its components. However, a thorough comparison with a broader range of machine learning and deep learning models remains an important direction for future research. Furthermore, the choice of evaluation metrics should reflect the specific goals of the groundwater level prediction task and model evaluation should also consider other metrics such as training time and memory usage to reach a performance level.

**Fig. 3:**
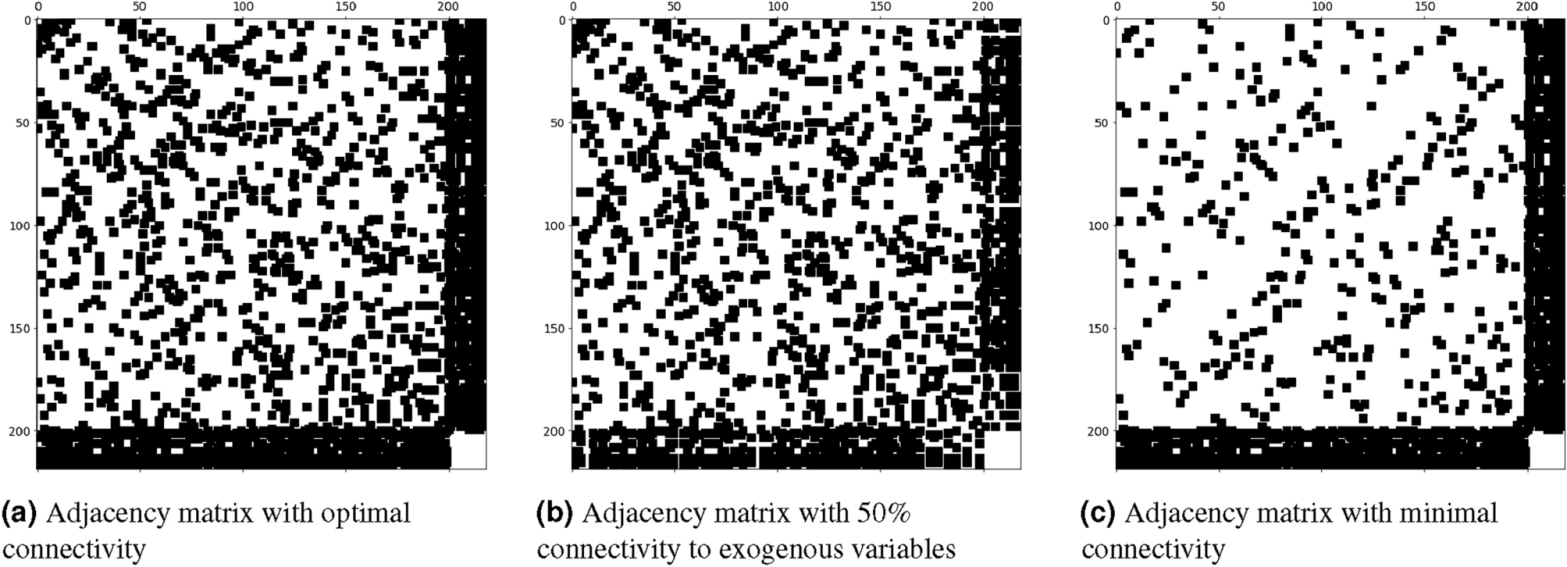
Comparison of adjacency matrices representing different levels of connectivity in the modeled groundwater network. The first 200 rows represent observation wells in random order, while the last 19 rows and columns represent exogenous variables. Figure 3a depicts the adjacency matrix corresponding to the best case scenario where each observation well is connected to its three nearest wells based on Euclidean distance. Additionally, connections extend to all pumping stations, the nearest precipitation and evaporation monitors, and the two proximal river measurement sites, explaining the increased density seen in the last 19 rows and columns beyond the 200 observation wells. Figure 3b and c illustrate matrices with reduced connectivity: the former with only half of the observation wells linked to exogenous variables, and the latter with each well connected to only its single nearest counterpart.

The network graph’s definition is also shown to be crucial for performance. The series of adjacency matrices depicted in Fig. 3 illustrates various connectivity levels among observation wells and exogenous variables. Figure 3a illustrates the proposed network configuration, establishing connections for each well to its three nearest counterparts, all pumping sites, the closest precipitation and evaporation monitoring stations, and the two nearest river gauging points. Within the adjacency matrix, the values assigned to these connections range from 0.1 to 0.5, reflecting the varying types of hydrological interactions. Conversely, setting all non-zero values in the matrix to 1 leads to a significant error increase of approximately 112.75%. This change highlights the critical role of detailed connectivity, affirming that this configuration yields a balanced and empirically validated network structure. The matrix’s denser lower segments reveal more intense connections with external variables. By contrast, Fig. 3b, which features only half of the observation wells-randomly selected-connected to exogenous variables, results in a more sparse matrix and an elevated RMSE of 15.0 cm. This underscores the importance of linking all observation wells to exogenous variables. The minimal connectivity scenario in Fig. 3c, where each well is linked to only its nearest neighbor, results in the sparsest matrix and an increased error of 10.3 cm. Similarly, connecting each well to four of its neighbors results in a higher error of 10.7 cm. Enabling the network to learn the adjacency matrix, as described by Wu et al.^14^, resulted in an higher error of 10.90 cm. In contrast, employing the predefined adjacency matrix with the proposed connectivity achieved a 25.4% improvement in RMSE compared to allowing the network to learn the adjacency matrix. This indicates that while the strategy of allowing the network to autonomously learn the adjacency matrix can be beneficial initially, it does not lead to the optimal outcome when compared to the adjustments made to address the deficiency.

The ablation study further evaluates the model’s sensitivity to a range of hyperparameters, encompassing those impacting both temporal and spatial convolutional layers, with the aim of optimizing forecast accuracy. In the finalized implementation of the ST-GNN model, the depth of the graph convolution is set to four layers, with kernel sizes selected as 1 and 2. To prevent overfitting, a dropout rate of 50% is implemented. Moreover, the model employs a $\beta$ value of 0.05 to preserve the original states of the root nodes.

Finally, the impact of the multi-step-ahead function on the model’s training efficacy is investigated. Drawing inspiration from the approach taken by^31^, the effect of extending the forecast horizon on model performance is examined. The results align with Bentivoglio et al.’s findings, showing that increasing the number of steps ahead consistently improves model accuracy. However, this enhancement comes with higher memory requirements and longer training times. Specifically, the duration needed for each training epoch increases from 2 seconds with a single forecast horizon to 93 seconds for 10 forecast windows, equating to a period of 10 weeks. An optimization analysis of the forecast horizon reveals that the model achieves notable performance within just a three-week timeframe. While further extension of the forecast horizon does yield improved outcomes, the rate of performance gain diminishes, nearly reaching a plateau beyond three weeks. Therefore, a six-week period is chosen for real-world data analysis in the following section to strike an optimal balance between enhancing performance and managing the increased memory demands associated with a longer forecast horizon. This strategy ensures peak performance while maintaining computational efficiency.

### Real-world data application

**Fig. 4:**
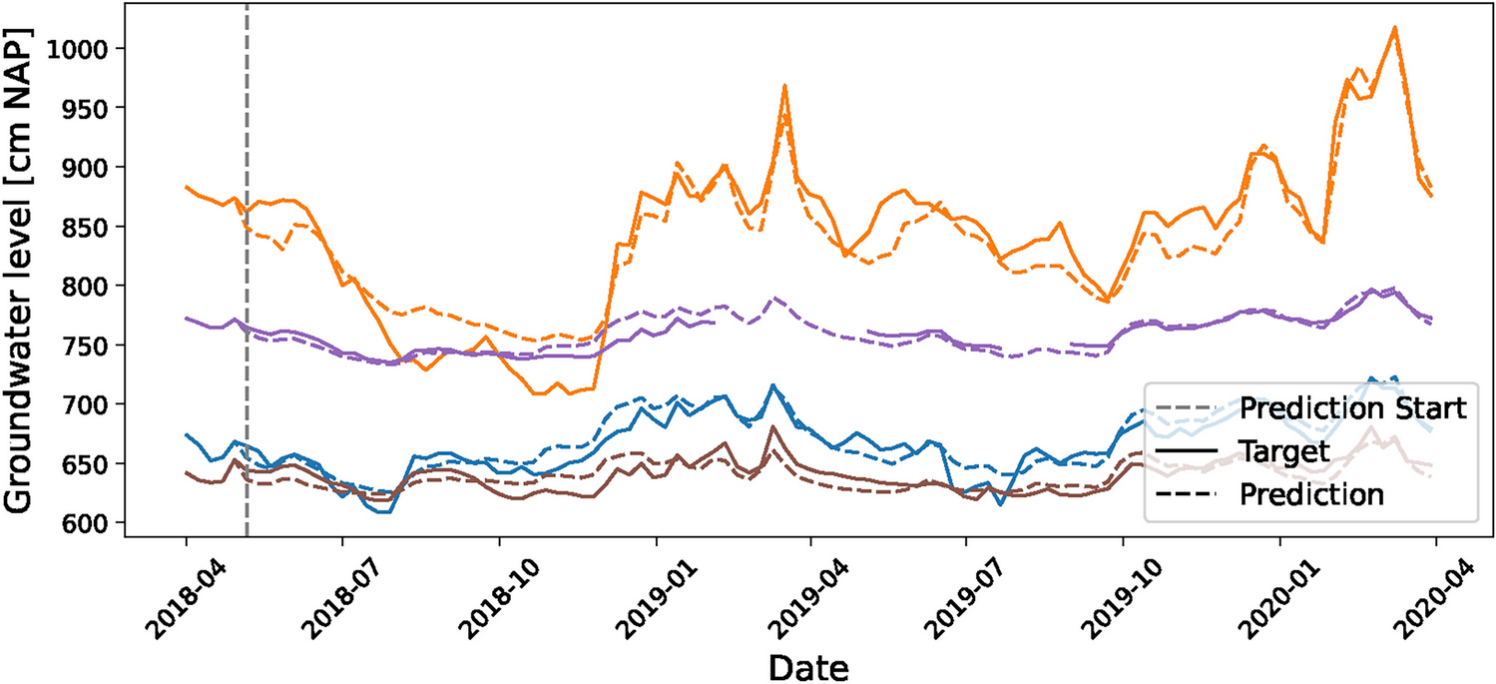
Comparative performance of the model’s predictions over four randomly selected piezometers. The model utilizes a past window of 5 weeks and extends its forecasts to demonstrate its proficiency in capturing groundwater level trends over a longer horizon. Solid lines correspond to observed data, while dashed lines represent forecasts.

This section examines the application of the deep learning model to real-world datasets. Figure 4 presents a comparison between forecasts for four randomly selected observation wells and actual data that was not seen by the model during training, beginning in April 2018. The solid lines represent the actual measured groundwater levels, while the dashed lines depict the model’s forecasts. This figure illustrates that the model closely aligns with the actual observed groundwater levels. Despite being trained to forecast up to 6 weeks ahead, the model demonstrates remarkable extrapolation capabilities for up to 100 weeks, as evidenced by its predictions, which closely follow the trends observed in the actual groundwater measurements without any significant error propagation or accumulation. In contrast to synthetic data, these real-world datasets include missing values. As indicated in the figure, where there is a prediction for a missing window in an observational well, it underscores the model’s ability to predict for periods where data are unavailable. This highlights another potential application of the model for data infilling.

**Fig. 5:**
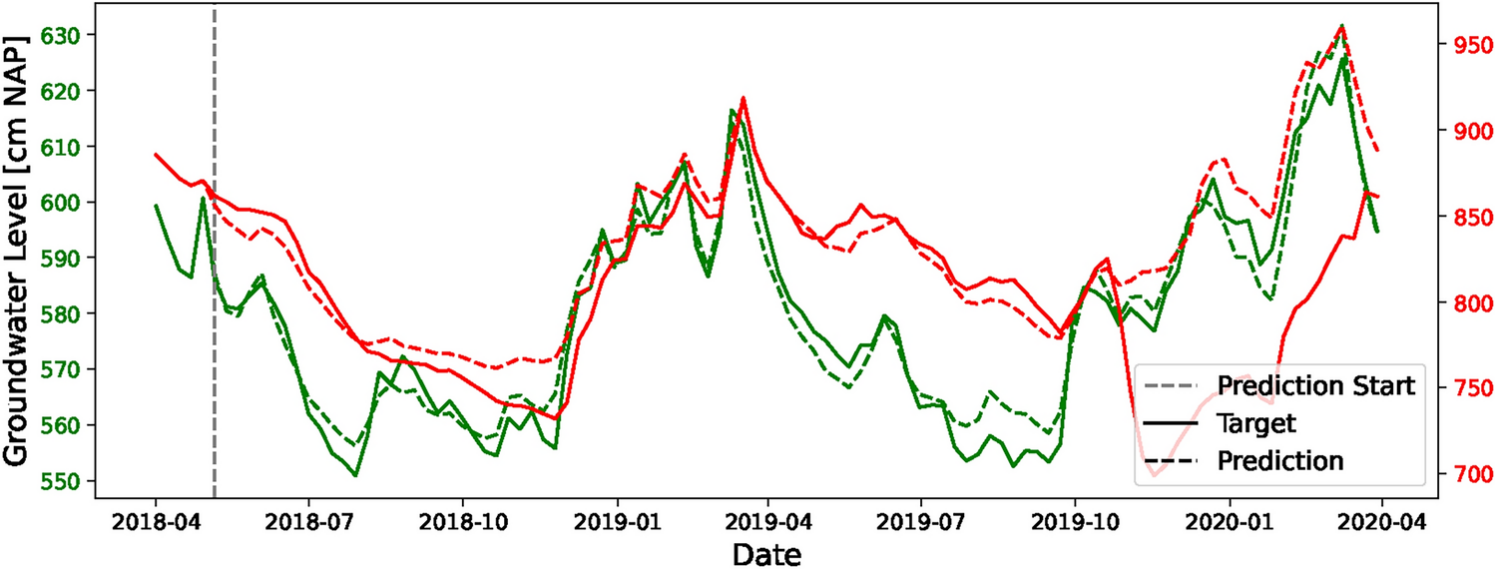
Comparative analysis of the model’s forecasting accuracy for observation wells, highlighting those with the highest (red) and lowest (green) RMSE. Solid lines indicate observed data, while dashed lines denote forecasts.

Figure 5 showcases two contrasting results, featuring selected wells with very high and very low RMSE values. The green lines depict the best testing outcome, which achieves an RMSE of 4.2 cm; this result closely follows the target trajectory with only minor prediction errors that do not result in error amplification. Conversely, the red line represents a test sample with an RMSE of 53.4. In this scenario, a larger variation in groundwater levels is observed; however, the model still manages to forecast within this wider range. Although the trend is predicted, there is a notable discrepancy in the values towards the end. This sample ranks as the second worst, surpassed only by another with an RMSE of 67.6 cm, which is not depicted here due to its relatively stationary trend. It is noteworthy that these worst two samples originate from the same site but at different depths, hinting at a potential issue within the system, possibly missing some external information. This suggests that the model could also serve as a tool for identifying such anomalies. Despite these findings, no specific pattern or geographical distribution of errors could be determined.

**Fig. 6:**
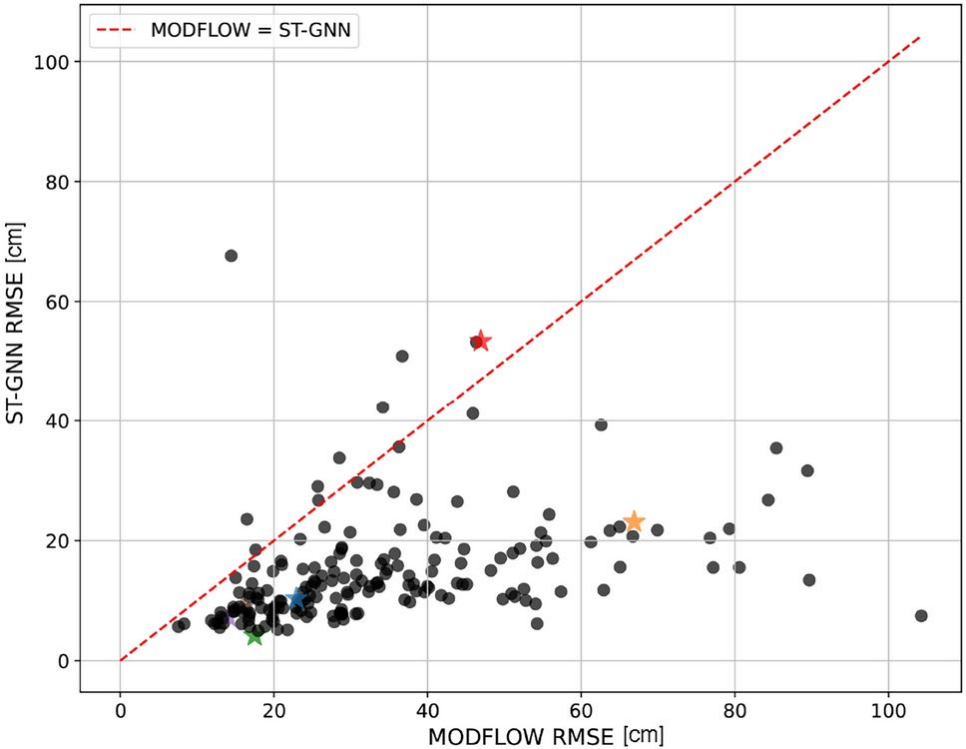
RMSE scatter plot comparison between the GNN and MODFLOW models, with the ST-GNN model predominantly achieving lower RMSE values. Stars and corresponding colors highlight the specific observation wells from Figs. 4 and 5..

When benchmarked with real data, the traditional MODFLOW model exhibits an RMSE of 25.2 cm, thus showcasing the enhanced precision of the deep learning model in the forecasting task. Figure 6 presents a scatter plot comparison of the RMSE values for the ST-GNN and MODFLOW models. In this plot, each data point corresponds to a unique piezometer in the test set, with the inclusion of an equality line serving as a reference to easily discern which model achieves a lower RMSE. Moreover, observation wells featured in Figs. 4 and 5 are marked with a star, with colors matching their depiction in the plots. Data points situated beneath the equality line indicate superior performance by the ST-GNN model. The aggregation of points below the equality line corroborates the ST-GNN model’s consistent outperformance, validating its efficacy in predicting groundwater levels. It is noted that, with the exception of the observation well with the highest error, all other high-RMSE predictions, such as the one visualized in Fig. 5, do not lie far from the equality line in Fig. 6.

In conclusion, evaluating the ST-GNN model on real-world data showcases its robustness and reliability. The model yields more accurate forecasts than traditional MODFLOW models, showcasing the profound potential of deep learning in groundwater level forecasting.

## Summary and conclusions

This study successfully applies Spatial-Temporal Graph Neural Networks (ST-GNNs) to predict groundwater levels in the Overbetuwe area, Netherlands, demonstrating their effectiveness in handling the complex nonlinearity and spatial-temporal dynamics of groundwater systems. The ST-GNN model significantly outperforms traditional numerical models like MODFLOW, particularly in long-term forecasting accuracy, and proves robust in managing missing data. By integrating a comprehensive dataset, including 395 groundwater level time series and auxiliary data, the model excels in capturing intricate interactions within the groundwater network. The graph-based framework, with its predefined adjacency matrix, further enhances model performance, as highlighted by an ablation study that underscores the importance of the temporal and graph convolutional modules.

Future research could focus on enhancing the model’s predictive accuracy by integrating additional variables into the model architecture, such as incorporating resistance as weights within the graph’s edges. Explicitly including features such as the top and bottom elevations of aquifers, their hydraulic conductivity, and the resistance in aquitards within the dataset could significantly improve the model’s performance. Moreover, the scalability of this approach to encompass larger networks of groundwater measurements and the integration of additional exogenous variables present promising avenues for research and application. The development of networks capable of processing multi-modal inputs, including continuous geological information, could further enhance the model’s predictive accuracy and utility. Importantly, by incorporating additional continuous variables, it would be interesting to develop a model that can extrapolate and predict also in points that are not observation wells, thereby truly competing with traditional models which currently provide solutions in the whole domain.

The application of ST-GNNs in groundwater level prediction not only demonstrates superior accuracy over traditional models but also offers significant potential for strategic water resource management and planning, making it a valuable tool for addressing future hydrological challenges. In summary, this study contributes significantly to advancing the application of machine learning in hydrology, establishing the potential for ST-GNNs to become a a powerful tool for predictive modeling of groundwater levels.

## Data Availability

The real-world data supporting the findings of this study, will be made available along with the associated code. However, the synthetic data, privately provided by Vitens and essential for our research, will not be freely available due to licensing requirements associated with users of the MORIA model. To request access to the data, please contact Maria Luisa Taccari at marialuisa.taccari@outlook.com.

## Code availability

All codes used to generate the datasets and train the model will be made available at https://github.com/mlttac/GroundwaterFlowGNN.
